# Optimization of Sacrificial Layer Etching in Single-Crystal Silicon Nano-Films Transfer Printing for Heterogeneous Integration Application

**DOI:** 10.3390/nano11113085

**Published:** 2021-11-16

**Authors:** Jiaqi Zhang, Yichang Wu, Guofang Yang, Dazheng Chen, Jincheng Zhang, Hailong You, Chunfu Zhang, Yue Hao

**Affiliations:** State Key Discipline Laboratory of Wide Band Gap Semiconductor Technology, School of Microelectronics, Xidian University, 2 South Taibai Road, Xi’an 710071, China; jqzhangxd@163.com (J.Z.); bingkongyanye@163.com (Y.W.); gfyang@stu.xidian.edu.cn (G.Y.); dzchen@xidian.edu.cn (D.C.); jchzhang@xidian.edu.cn (J.Z.); hlyou@mail.xidian.edu.cn (H.Y.); yhao@xidian.edu.cn (Y.H.)

**Keywords:** single-crystal silicon nano-films, transfer printing, heterogeneous integration, sacrificial layer, Si MOSFET

## Abstract

As one of the important technologies in the field of heterogeneous integration, transfer technology has broad application prospects and unique technical advantages. This transfer technology includes the wet chemical etching of a sacrificial layer, such that silicon nano-film devices are released from the donor substrate and can be transferred. However, in the process of wet etching the SiO_2_ sacrificial layer present underneath the single-crystal silicon nano-film by using the transfer technology, the etching is often incomplete, which seriously affects the efficiency and quality of the transfer and makes the device preparation impossible. This article analyzes the principle of incomplete etching, and compares the four factors that affect the etching process, including the size of Si nano-film on top of the sacrificial layer, the location of the anchor point, the shape of Si nano-film on top of the sacrificial layer, and the thickness of the sacrificial layer. Finally, the etching conditions are obtained to avoid the phenomenon of incomplete etching of the sacrificial layer, so that the transfer technology can be better applied in the field of heterogeneous integration. Additionally, Si MOSFETs (Metal-Oxide-Semiconductor Field Effect Transistors) on sapphire substrate were fabricated by using the optimized transfer technology.

## 1. Introduction

In the past few decades, under the guidance of Moore’s Law, the minimum size of silicon-based electronic devices has gradually decreased, and the performance and computing speed of circuits have continuously increased. However, due to the physical limitations, in the foreseeable future, the size of silicon-based devices can no longer continue to shrink and meet the Moore’s Law [1]. At this time, single-crystal silicon nano-films and compound semiconductor materials have attracted widespread attention due to their unique advantages. Single-crystal silicon nano-films have unique optical and thermal properties that are different from silicon bulk materials. Compound semiconductors represented by GaN materials have advantages such as higher breakdown voltage and electron mobility. Therefore, it is extremely urgent to integrate monocrystalline silicon devices and compound semiconductor devices through heterogeneous integration to meet the development requirements of ultra-miniaturization, intelligence, and diversification of electronic systems, and to break through the limitations of silicon bulk materials. There are several methods in the current stage of heterogeneous integration technology, such as epitaxial growth, bonding, three-dimensional packaging, and transfer printing [2,3,4,5,6,7,8,9]. This article mainly studies the transfer technology applied in the field of heterogeneous integration. Compared with other heterogeneous integration technologies, transfer technology has many advantages. First of all, the transfer technology can be performed at room temperature, avoiding the adverse effects that high and low temperature environments may have on the function of the device. Secondly, the transfer technology has strong compatibility. The transfer object can be simple nanowires, two-dimensional structures, or even complex three-dimensional structures [10,11,12,13,14]. This makes the transfer technology compatible with many other processing technologies. Finally, the transfer technology also has the advantages of low cost and simple operation. Combined with the unique advantages, transfer technology has huge application potential in many fields.

However, in the process of using the transfer technology for single-crystal silicon nano-films, it is found that the process of wet etching the sacrificial layer (SiO_2_ layer) in the transfer technology often fails to achieve the expected effect, and there is a phenomenon that the SiO_2_ layer cannot be completely etched. As a result, the subsequent transfer process and the preparation of the device cannot be carried out [15,16]. Therefore, this article explores the different conditions of the size of Si nano-film on top of the SiO_2_ layer, the location of the anchor point, the shape of Si nano-film on top of the SiO_2_ layer, and the thickness of the SiO_2_ layer that affect the process of wet etching the SiO_2_ layer, and analyzes the principle of incomplete etching of the SiO_2_ layer. Through the controlled variable experiment, the optimal value range of each condition in the process of etching the SiO_2_ layer is selected, so as to avoid the phenomenon of incomplete etching of the SiO_2_ layer.

## 2. Experiment Process

**Figure 1:**
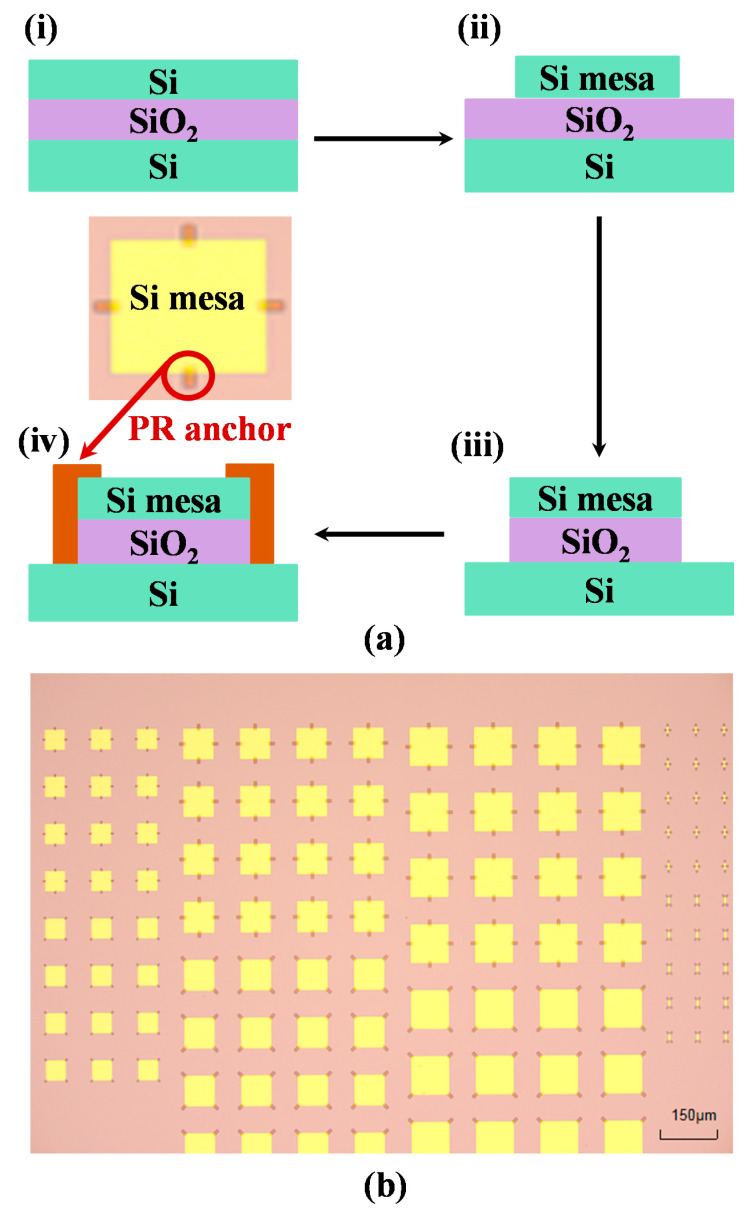
(**a**) The main process steps of SOI substrate pretreatment, (**b**) SOI substrate optical photo obtained after the pretreatment.

There are two types of the single-crystal silicon nano-film donor substrates. One is a 200/200 nm SOI wafer (Soitec Bernin, France by Smartcut with 200 nm top Si (100), which is doped boron. And, the doping concentration is 8 × 10^16^ cm^−3^). The other is an 145/100 nm SOI wafer (Soitec by Smartcut with 145 nm top Si (100), which is doped boron, whose level is 8 × 10^16^ cm^−3^ and 100 nm buried oxide). In this study, the main process steps of the SOI substrate pretreatment are shown in Figure 1a. First, SOI substrates of 2 cm × 3 cm were selected as the donor substrates, as shown in Figure 1(ai). The substrates were ultrasonically cleaned in acetone, absolute ethanol, and water. Then, the substrates were soaked in the piranha solution (H_2_SO_4_:H_2_O_2_ = 3:1) for 20 min. Lithography and RIE were used to make various Si mesas of different sizes and shapes, as shown in Figure 1(aii). Table 1 shows the specific shapes and sizes of Si mesas. Subsequently, the substrates were immersed in DHF (dilute hydrofluoric acid) (1:50) for 50 min to remove the exposed BOX (buried oxide) around the Si mesas, as shown in Figure 1(aiii). After that, PR (photoresist) anchors were formed by photolithography, as shown in Figure 1(aiv). These PR anchors are a photoresist pattern formed by lithographic, and it can prevent the Si mesas from scattering or moving when the BOX is completely removed by HF [7,8,17]. The substrates obtained through the above process are shown in Figure 1b. Finally, these prepared SOI substrates through pretreatment process are immersed in 20% and 40% HF to remove the BOX-layer under Si mesas for a variety of comparative experiments, respectively.

## 3. Results and Discussion

The comparison experiments on the influence of the size of Si nano-film on top of the BOX layer, PR anchors’ position, the shape of Si nano-film on top of the BOX layer, and the thickness of the sacrificial layer on the sacrificial layer etching are carried out. Incomplete etching of the sacrificial layer is mainly caused by two factors, namely surface tension and the electrical double layer effect. These factors cause the top Si mesas to adhere to the bottom Si substrate during the wet etching process. These factors prevent HF from getting underneath the Si nano-film and reaction product from removing from this position, causing the etching process to stop.

During the wet etching process, there is a partly suspended structure composed of the top Si nano-film, HF etching solution, and bottom Si substrate. Due to the surface tension, there is a kind of “collapse” tendency of parallel flat plates formed by unbalanced molecular cohesion on or near the surface [6,18,19]. The vertical liquid surface shows a “collapse” phenomenon, which prevents the liquid from further squeezing into the interior. Additionally, the “collapse” phenomenon can be characterized by the pull-up length. It is an important criterion for judging whether the BOX layer etching can be completely removed from underneath the silicon nano-film. The pull-up length refers to the maximum length of the microstructure without physical contact with the substrate under the condition of no external force and static action. The specific formula is as follows [17,20]:

(1) $$L=1.059{\left[\frac{Ed^{2}h^{3}}{2\gamma \left(1-\upsilon^{2}\right)\left(cos\theta_{1}+cos\theta_{2}\right)}\right]}^{\frac{1}{4}}\;$$

Among them, “*E*” is the elastic modulus of the structured film. For the top Si nano-film in this work, the value range of “*E*” is 130 GPa to 188 GPa. “*υ*” is the Poisson’s ratio of the top Si nano-film, and the value range in this experiment is 0.064 to 0.28. “*γ*” is liquid surface tension. “*d*” is the thickness of the sacrificial layer. “*h*” is the thickness of the structural film. Additionally, “$\theta_{1}$”, “*θ*_2_” are the solid-liquid contact angles. Two types of SOI substrates are used in this work, Si/SiO_2_ = 200/200 nm, 145/100 nm, respectively. “*L*” obtained by substitution into Formula (1) is 13.0 μm and 7.2 μm, respectively.

**Figure 2:**
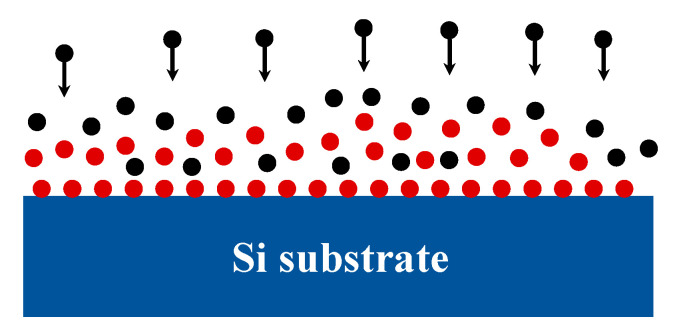
Electrical double layer model. The red balls represent positive ions, and the black balls represent counter ions.

The second factor is the electrical double layer effect [20]. There will be static charges on most solid surfaces, as shown in Figure 2. The red balls represent positive ions which will attract counter ions (the black balls) in the solution (HF), causing particles to accumulate in wet etching channels. This phenomenon will prevent the further diffusion of hydrofluoric acid and form a diffusion limit. The thickness of the electrical double layer can be calculated according to the Poisson-Boltzmann equation. The formula is as follows [20]:

(2) $$\beta ={\left(\frac{2n_{i0}z^{2}e^{2}}{\varepsilon \varepsilon_{0}kT}\right)}^{\frac{1}{2}}$$

In this formula, “(*β*)^−1^” is the thickness of the electrical double layer; “*ε*_0_” is the vacuum dielectric constant; “*ε*” is the dielectric constant in the medium; “*z*” is the valence of the i-th ion; “*k*” is the Boltzmann constant; “*T*” is the absolute temperature of the colloidal solution; “*e*” is the electronic charge; and “*n_i0_*” is the volume concentration of the i-th ion. Combining the above formula can calculate the thickness of the electrical double layer in these experiments. At room temperature, 40% HF (22.6 mol/L) was used for wet etching of buried oxide under Si mesas. Since HF is not completely ionized in water, its ionization constant is A = 3.53 × 10^−4^. The calculated concentration of hydrogen ions and fluoride ions is about 0.0080 mol/L, and the calculated “(*β*)^−1^” is 2.73 nm at room temperature. The thickness of the electrical double layer is affected by temperature and ion concentration. It increases with the increase of temperature and decreases with the increase of ion concentration. In the same way, the thickness of the electrical double layer is 3.93 nm in 20% hydrofluoric acid.

In the following subsections, influence of the size and shape of Si nano-devices on the sacrificial layer wet etching is studied, as well as the influence of the location of the PR anchor point and the thickness of the BOX layer.

### 3.1. Influence of Graphic Size of the Top Si Mesa on Wet Etching Effect

**Figure 3:**
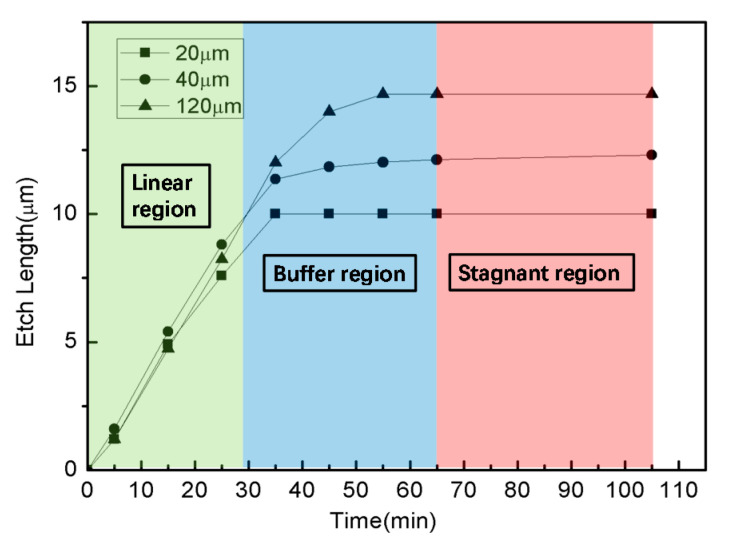
The relationship between the etching time and the transverse etching length of the sacrificial layer of differently sized square Si mesas in 20% HF.

The top Si mesas are often designed to different sizes and shapes according to design requirements. The square Si mesas on the pretreated SOI substrate (Si/SiO_2_ = 200/200 nm) were immersed in HF (20%) for etching the sacrificial layer under Si mesas. The sides of the square Si mesas are 20 μm, 40 μm and 120 μm, respectively. The relationship between the etching time and the transverse etching length of the square top Si mesas with side lengths of 20 μm, 40 μm and 120 μm is obtained, and shown in Figure 3. The transverse etching length is defined as the length from the edge of the top Si mesa to the edge of the unetched buried oxide layer. It can be seen from Figure 3 that the etching rate of the sacrificial layer in HF is constant up to 30 min, and the transverse etching length has a linear relationship with the etching time. This process is named the linear region. From 30 to 65 min, the etching rate of the sacrificial layer gradually decreases, which is called the buffer region. After the 65 min, the etching of sacrificial layer by HF stops, and the transverse etching length does not change. This process is called the stagnant region in our work. In this experiment, only the buried oxide layer of the Si mesa with the side length of 20 μm is etched completely (S_20_ = 10 μm < L = 12.98 μm, “S_20_” stands for half side length of the square Si mesa whose side length is 20 μm); that is, these 20-μm-sized mesas can be transferred, while the other two sizes of silicon mesas cannot because the length of the BOX area to be removed is too large (S_40_ = 20 μm > L = 12.98 μm, S_120_ = 60 μm > 12.98 μm). Therefore, the size of the Si mesa should not be too large, otherwise the bottom cutting cannot be completed, resulting in the inability to transfer printing.

### 3.2. Influence of the Location of the PR Anchors on Wet Etching Effect

**Figure 4:**
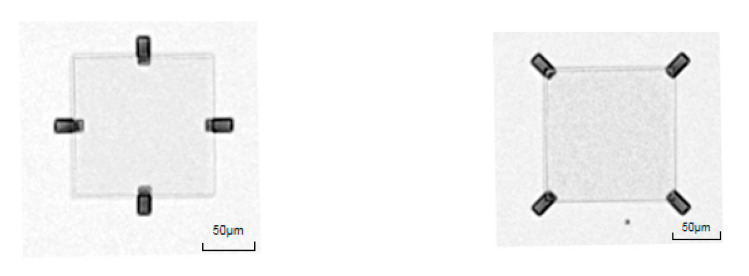
Two representative locations of the PR anchors.

**Figure 5:**
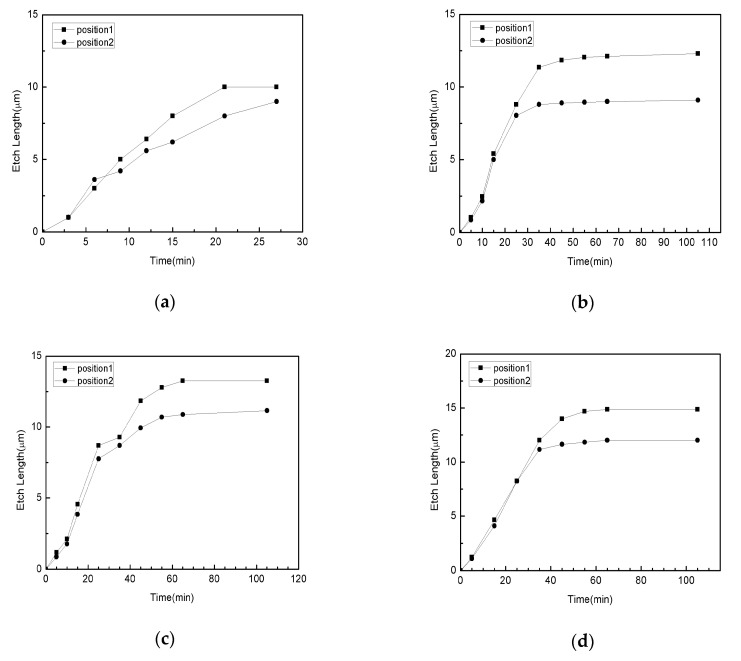
The relationship between the etching time and the transverse etching length of the sacrificial layer with the same size and shape using two PR anchors. (**a**) Square with a side length of 20 μm, (**b**) square with a side length of 40 μm, (**c**) square with a side length of 80 μm, (**d**) square with a side length of 120 μm.

The positions of the PR anchors have an important effect on the etching rate of the sacrificial layer and the transverse etching length. The PR anchors play a role in fixing Si mesas so that Si mesas will not drift or fall off during the wet etching process. In this work, two representative locations of the PR anchors are used, as shown in Figure 4. The PR anchor points have the same size, 5 μm × 15 μm. The first set of the PR anchors is distributed along the middle of the four sides of the Si mesa (position 1), and the second set of the PR anchors are positioned at the four corners of the Si mesa (position 2). The pretreated SOI substrate was soaked in 40% HF. The relationship between the transverse etching length and etching time of the Si mesas with side lengths of 20 μm, 40 μm, 80 μm and 120 μm is shown in Figure 5. As can be seen for 20 μm square mesas, the position of the anchor point does not matter; both sets show release of the mesas. Similar to the 20 μm square mesas, the 40 μm square Si mesas have different transverse etching lengths. The stagnant transverse etching lengths of “position 1” and “position 2” are 12.3 μm and 9.0 μm, respectively. For the square Si mesas with the size of 80 μm, the stagnant transverse etching length of “position 1” is 13.3 μm, and the transverse etching length of “position 2” is 11.2 μm. For the square Si mesas with the size of 120 μm, the transverse etching length of “position 1” is 14.9 μm, and the transverse etching length of “position 2” is 12.0 μm. Clearly, the position of the PR anchors in the middle of the four sides of the Si mesa is better for etching the sacrificial layer. This is because the length of the edge of the Si mesa covered by the second type of PR anchor (position 1) is longer than that of the first type of PR anchor (position 2), so that the contact area between the sacrificial layer and HF becomes smaller with the second type of PR anchor, which is not conducive to the etching of sacrificial layer by HF.

### 3.3. Influence of Graphic Shape of the Top Si Mesa on Wet Etching Effect

**Figure 6:**
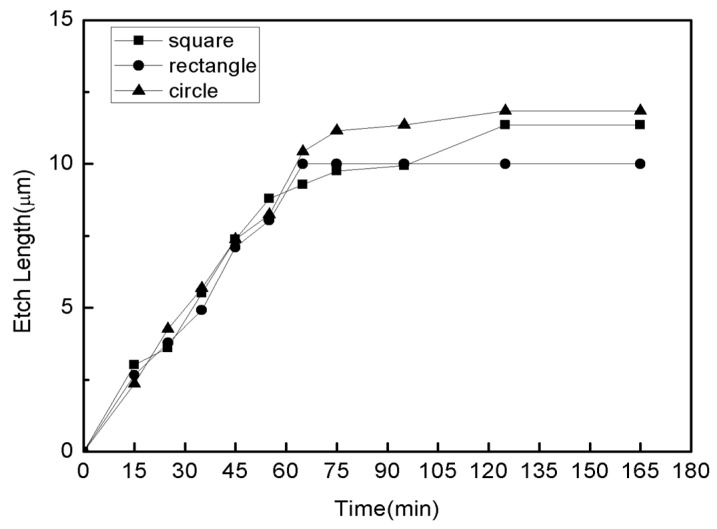
The relationship between the etching time and the transverse etching length of sacrificial layers underneath three types of Si mesas with identical circumference lengths but different shapes.

**Figure 7:**
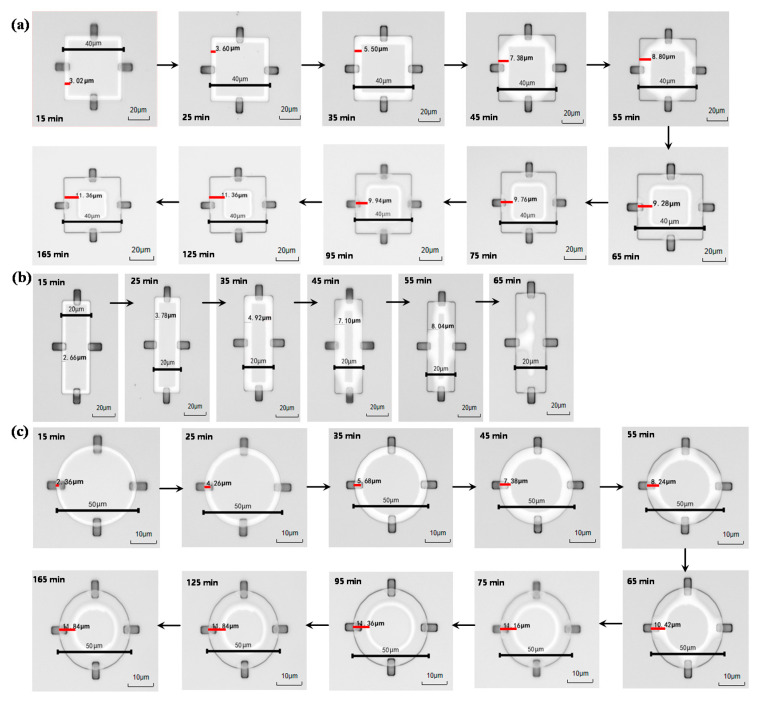
Optical images of the sacrificial layer etching process; (**a**) square sacrificial layer with side length of 40 μm, (**b**) rectangular mesas with a width and a length of 20 μm × 60 μm, and (**c**) circular mesas with a diameter of 50 μm.

In this experiment, three different Si mesa shapes on top of sacrificial layers were fabricated, i.e., square, rectangular and circular Si mesa, respectively. A comparison between three with a fixed perimeter of 160 μm (the perimeter is the circumference of the Si mesa) is done to ensure the same contact area between the etching front and the hydrofluoric acid. It can be seen from Figure 6 that the transverse etching rates of the three shapes are not much different, but in the end only the sacrificial layer under rectangular mesas is completely etched, whereas the sacrificial layer underneath square and circular mesas are both incompletely etched, as shown in Figure 7. The complete etching process of three shapes is shown in Figure 7. When the BOX between the top Si nano-film and Si substrate was etched completely by HF, the top Si nano-film would stick to the Si substrate. And, it shows the white spots in the sticky regions. Only the rectangular sacrificial layer shape is completely etched. This is because the shortest side of the rectangular sacrificial layer is less than twice the calculated pull-up length. In this direction, the electrical double layer effect during the etching process will not affect the etching process before the release is completed.

### 3.4. Influence of Thickness Ratios of Si/SiO_2_

**Figure 8:**
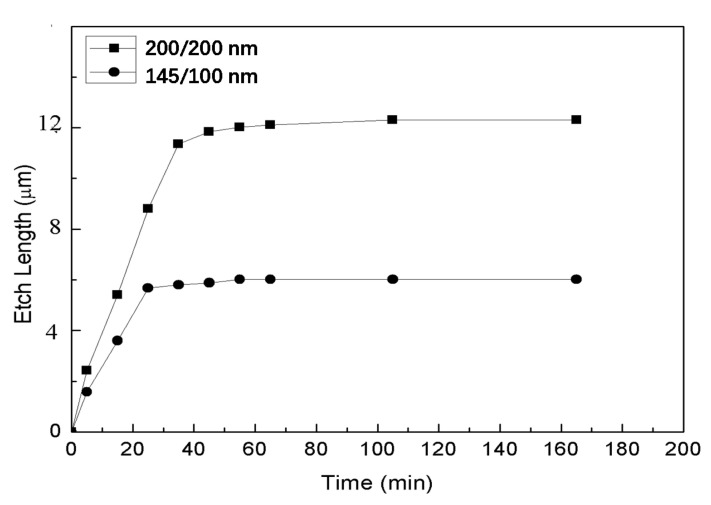
The relationship between the etching time and the transverse etching length of the SiO_2_ sacrificial layer underneath a square mesa.

Two SOI substrates with different thickness ratios of Si/SiO_2_ were used. These two kinds of thickness ratios of Si/SiO_2_ were 200/200 nm and 145/100 nm, respectively. Under the same other conditions, the square Si mesas with side length of 20 μm were compared. The relationship between the etching time and the transverse etching length of the square sacrificial layer with different thickness ratio of Si/SiO_2_ is shown in Figure 8. For the sacrificial layer with Si/SiO_2_ = 145/100 nm, the transverse etching rate of the sacrificial layer is lower than that of Si/SiO_2_ = 200/200 nm. In addition, the transverse etching length of the sacrificial layer with Si/SiO_2_ = 200/200 nm is longer than that of the sacrificial layer with Si/SiO_2_ = 145/100 nm. Finally, in the comparison experiment of the sacrificial layer thickness, it can be found that the etching rate of the substrate with the sacrificial layer thickness of 100 nm is lower. This is mainly due to the fact that the thinner the thickness of the sacrificial layer, the greater the impact of the electrical double layer effect on the etching process. Due to the limited experimental conditions, we can only compare two kinds of thickness ratios of Si/SiO_2_, which is not complete, and a systematic study would be required to draw a safe conclusion.

### 3.5. Si MOSFET Transferred on Sapphire Substrate by Transfer Printing

**Figure 9:**
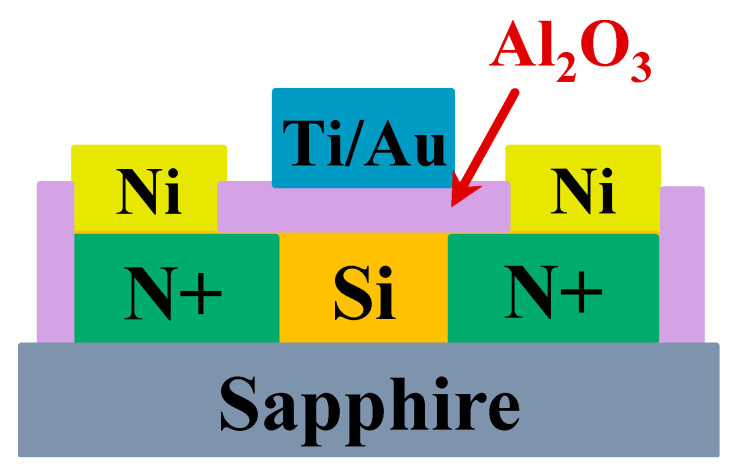
Diagram of the cross-section of Si MOSFET (Metal-Oxide-Semiconductor Field Effect Transistor) transferred onto sapphire substrate by transfer printing.

Figure 9 shows the diagram of the cross section of a Si MOSFET (Metal-Oxide-Semiconductor Field Effect Transistor) transferred on a sapphire substrate by transfer printing. SOI (Si/SiO_2_ = 200/200 nm) substrate was selected as the donor substrate. The preparation method of Si inks to be transferred was detailed in the above experimental process. The transfer printing and device fabrication process are described below. First, thermal released tape (TRT) was coupled to the donor substrate, and then quickly torn, allowing TRT to obtain the Si inks. TRT for obtaining the Si inks was coupled to the receiver substrate (sapphire). Then, the coupling system was placed on a hot plate at 125 °C to release the Si inks onto the sapphire substrate. Phosphorus ions were injected into the source and drain region, and then the impurity diffusion was activated for 60 s at 1000 °C by an RTP process. Twenty nanometers Al_2_O_3_ grown by ALD (Atomic layer deposition) served as the gate dielectric layer of Si devices. Then, 20/120 nm Ti/Au was deposited as gate metal electrodes of Si devices. Thirty nanometers Ni was deposited as the S/D electrodes of the Si devices, and then alloyed at 300 °C for 5 min. In this way, the preparation of the Si MOSFETs was completed.

**Figure 10:**
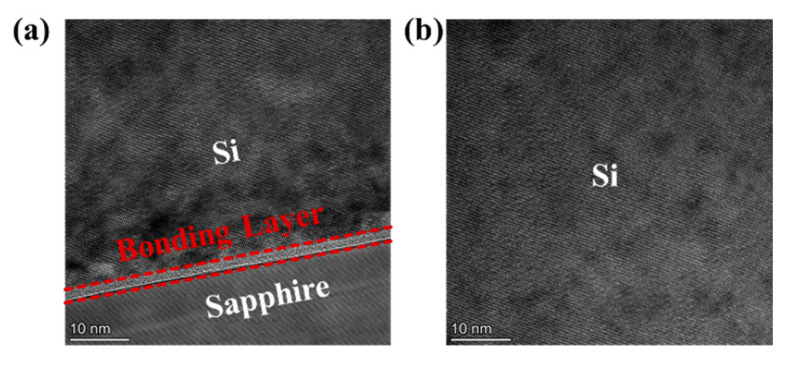
Diagram of the cross-section of (**a**) the Si/sapphire interface, and (**b**) Si nano-film.

Figure 10a shows the cross-section of the Si/sapphire interface of Si MOSFETs transferred onto sapphire substrate. The silicon bonded well with the sapphire. Due to the different lattice spacing between silicon and sapphire, the silicon near the bonding interface was subjected to lattice stress and became polycrystalline. Figure 10b shows the cross section of Si nano-film transferred on sapphire substrate. The lattice is neatly arranged without defects, thus transfer printing had no adverse effects on Si nano-film.

**Figure 11:**
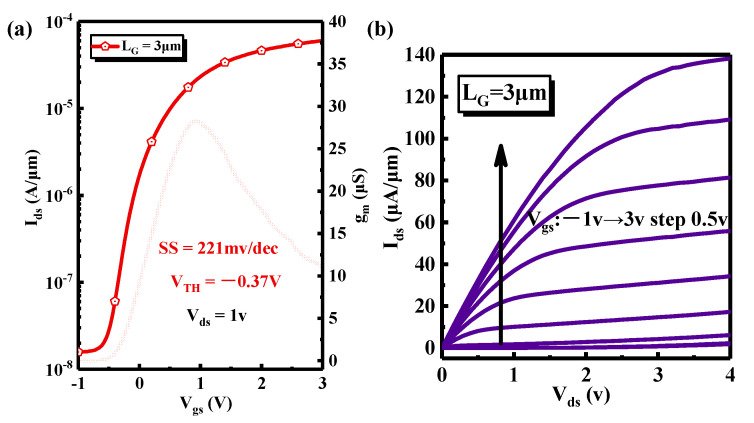

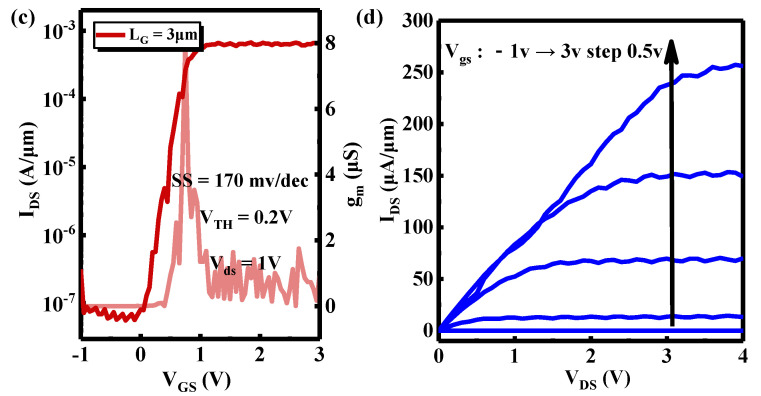
(**a**) The transfer characteristics of Si MOSFETs transferred on sapphire, (**b**) I-V characteristics of Si MOSFETs transferred on sapphire, (**c**) the transfer characteristics of the conventional Si MOSFETs, and (**d**) I-V characteristics of the conventional Si MOSFETs.

The transfer characteristics of Si MOSFETs are shown in Figure 11a. The gate length of the Si device is 3 μm, and the threshold voltage is −0.37 V. The peak transconductance is 30 μS and the subthreshold swing is 221 mV/dec. I-V characteristics of Si MOSFET (L_G_ = 3 μm) are shown in Figure 11b. The saturated output current can reach 140 μA/μm. For comparison, the characteristics of conventional Si MOSFETs are shown in Figure 11c. The gate length of the conventional Si MOSFETs is 3 μm, and the threshold voltage is 0.2 V. The peak transconductance is 8 μS and the subthreshold swing is 170 mV/dec. I-V characteristics of the conventional Si MOSFET (L_G_ = 3 μm) is shown in Figure 11d. The saturated output current can reach 254 μA/μm. Although the transferred Si film does not introduce defects in terms of TEM (FEI Tecnai G2 F20 transmission electron microscope instrument, Hillsboro, OR, USA) results, the device performances are not as good as that of conventional Si devices. There are two reasons; firstly, impurities were introduced in Si during transfer printing process. Secondly, the stress defect was introduced during RTP bonding. These reasons lead to the decrease of the device’s mobility and output current. We will optimize and improve transfer printing method and bonding technology to reduce defects and improve device performance.

## 4. Conclusions

This article analyzes the influence of the size of Si nano-film on top of the sacrificial layer, the location of anchor points, the shape of Si nano-film on top of the sacrificial layer, and the thickness of the sacrificial layer on the etching process. Through this article, we can have a deeper understanding of the principle and process of the wet etching process of this SiO_2_ sacrificial layer. In order to avoid incomplete etching of SiO_2_ layer, the etching conditions can be selected as follows: a SOI substrate with a larger sacrificial layer thickness should be chosen. Additionally, the sacrificial layer should be designed as a rectangle to ensure that the shorter side length is less than twice the pull-up length. The anchor points should be placed in the middle of the four sides of the sacrificial layer graphics. The use of this etching condition in the transfer technology can avoid the occurrence of incomplete etching of the sacrificial layer, which promotes the better application of the transfer technology in the field of heterogeneous integration. Based on the above results, Si MOSFETs transferred onto sapphire substrate were fabricated, and its I-V characteristics were good.

## Figures and Tables

**Table 1:** The shapes and sizes of Si mesas.

Square (The Length of its Sides)	Circle (The Radius)	Rectangle (The Length and Width)
20 μm	12.5 μm	30 μm, 10 μm
40 μm	25 μm	60 μm, 20 μm
80 μm	50 μm	120 μm, 40 μm
120 μm	75 μm	160 μm, 80 μm

## Data Availability

The data is not available due to further study.
